# Factors Associated with HBsAg Seropositivity among Pregnant Women Receiving Antenatal Care at 10 Community Health Centers in Freetown, Sierra Leone: A Cross-Sectional Study

**DOI:** 10.3390/pathogens11020243

**Published:** 2022-02-12

**Authors:** Manal Ghazzawi, Peter B. James, Samuel P. Massaquoi, Sahr A. Yendewa, Robert A. Salata, George A. Yendewa

**Affiliations:** 1 KnowHep Foundation, Freetown, Sierra Leone; knowhepfoundation.sl@gmail.com; 2 Faculty of Health, Southern Cross University, Lismore, NSW 2480, Australia; peter.james@scu.edu.au; 3 College of Medicine and Allied Health Sciences, University of Sierra Leone, Freetown, Sierra Leone; 4 Ministry of Health and Sanitation, Freetown, Sierra Leone; drspem@gmail.com (S.P.M.); syendewa@gmail.com (S.A.Y.); 5 Department of Medicine, Case Western Reserve University School of Medicine, Cleveland, OH 44106, USA; robert.salata@uhhospitals.org; 6 Division of Infectious Diseases and HIV Medicine, University Hospitals Cleveland Medical Center, Cleveland, OH 44106, USA; 7 Johns Hopkins Bloomberg School of Public Health, Baltimore, MD 21205, USA

**Keywords:** hepatitis B infection, pregnancy, antenatal screening, Sierra Leone

## Abstract

Hepatitis B (HBV) is a major public health threat in Sierra Leone. Pregnant women are disproportionately impacted, yet little is known about the epidemiology of HBV in this group. We conducted a cross-sectional study of pregnant women aged ≥16 years receiving antenatal care across 10 community health centers in Freetown from July to September 2021 to assess the prevalence and associated factors of HBsAg seropositivity. Logistic regression was used to identify the predictors of HBsAg seropositivity. In total, 394 pregnant women were screened. The mean age was 24.4 ± 4.9 years, 78.2% were married, and 47.2% were in the second trimester. Only 1% had received the HBV vaccine. The prevalence of HBsAg was 7.9%, while HIV was 5.8% and HIV/HBV co-infection was 0.3%. Regarding high-risk practices, 76.6% reported female genital circumcision, 41.9% ear piercing, 29.0% endorsed multiple sexual partners, and 23.6% reported sexually transmitted infections. In the logistic regression analysis, having a husband/partner with HBV (adjusted odds ratio (aOR): 6.54; 95% CI: [1.72–24.86]; *p* = 0.006) and residing in Central Freetown (aOR: 4.00; 95% CI: [1.46–11.00]; *p* = 0.007) were independently associated with HBsAg seropositivity. Our findings support the scaling up of HBV services to target pregnant women and their partners for screening and vaccination to help reduce mother-to-child transmission rates in Sierra Leone.

## 1. Introduction

Hepatitis B virus (HBV) poses a major public health threat globally. Recent reports have indicated that there are an estimated 296 million people chronically infected with HBV (CHB) [1]. Sub-Saharan Africa (SSA) has the second highest burden of HBV, with 6.1% of the general population affected by CHB [1]. The clinical consequences of untreated CHB are devastating, with 1 million people estimated to prematurely die annually from cirrhosis, end-stage liver disease and hepatocellular carcinoma [1]. Consequently, in 2015, the World Health Organization (WHO) launched an ambitious global agenda aiming to eliminate HBV and the other hepatitis viruses as a public health threat by the year 2030 [2].

HBV is transmitted horizontally via infected bodily fluids, as with sexual contact, transfusion of contaminated blood products and sharps or needlestick injury, or vertically through mother-to-child transmission (MTCT) or perinatally [3]. In hyperendemic regions (defined as HBsAg prevalence ≥ 8% in the general population [1,4]), HBV is most frequently acquired through MTCT or through close contact during the early years of life [1,3]. The probability of establishing chronic infection is inversely related to the age at which the initial infection was acquired, with >90% of neonatal/infant infections and <5% of infections in children aged >5 years and adults resulting in chronic HBsAg carriage [1,4]. The risk of CHB is further amplified in neonates born to mothers with positive hepatitis B e antigen (HBeAg) serostatus [5]. Furthermore, infection acquired during the early years of life is associated with accelerated progression to cirrhosis, hepatic decompensation and hepatocellular carcinoma, underscoring the critical importance of the prevention of vertical transmission through universal antenatal screening programs [6].

Sierra Leone is a low-income West African country with an estimated population of 8 million people [7]. Although there are currently no nationally representative studies, Sierra Leone is regarded as a hyperendemic country, with an estimated countrywide HBV seroprevalence rate of 8% to 10% [8,9]. Pregnant women appear to be disproportionately impacted by the HBV epidemic, yet few studies have described the epidemiology of HBV in this vulnerable group in Sierra Leone [10]. A handful of small studies conducted among pregnant women in Sierra Leone have reported HBsAg seroprevalence rates ranging from 6% to 18.8% [10,11,12]. Women living with the human immunodeficiency virus (HIV) in this setting appear to have a 2-fold higher risk of HBsAg carriage compared with the general population [10]; however, to date, no study has undertaken an exhaustive analysis of the correlates of HBV infection among pregnant women in the country.

As a major step in the direction of controlling the HBV epidemic in Sierra Leone, the Ministry of Health and Sanitation introduced the HBV vaccination in the Expanded Program of Immunization (EPI) schedule for children in 2009 [10,13]. This policy action was also in line with the 2019 recommendation from the World Health Organization (WHO), which called for universal prenatal HBV screening and the vaccination of non-immune or exposed newborns and adults [14]. The administration of the monovalent birth dose (given within 24 h of birth) followed by at least two additional scheduled doses is >95% effective at preventing perinatal transmission of HBV [14,15]. However, Sierra Leone is yet to introduce the birth dose, as the current vaccination schedule starts at two weeks after birth [8,9,10]. Additionally, HBV vaccination coverage rates for children and catch-up vaccination for adults including pregnant women have remained low nationally, and the overall impact of the immunization program on reducing MTCT rates remains unclear [13].

**Figure 1:**
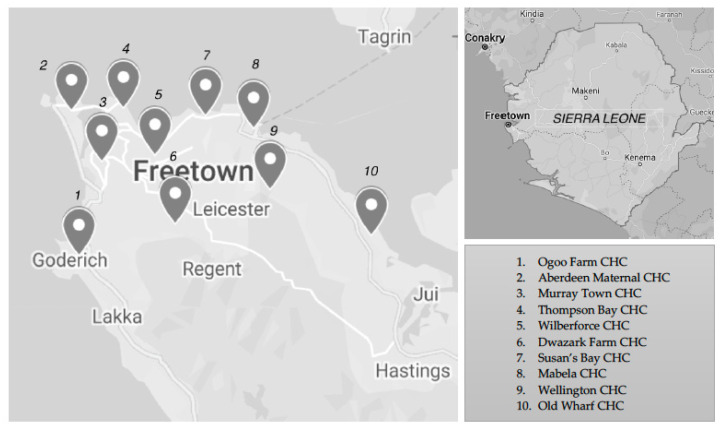
Community health center (CHC) locations in Freetown, Sierra Leone [16]. Abbreviations: CHC, community health center.

In order to ensure the effective prevention of MTCT (PMTCT) programs in Sierra Leone and countries with similarly high endemicity in SSA, it is essential to first assess the burden and clinical impact of HBV among pregnant women, especially in high transmission urban areas where they may be most vulnerable to the consequences of the disease. In this study, we utilized the resources of KnowHep Foundation Sierra Leone, a local non-governmental organization dedicated to raising awareness about viral hepatitis, to assess the prevalence and associated factors of HBsAg seropositive status among pregnant women receiving antenatal care across 10 community health centers (CHCs) (Figure 1) in Freetown, the capital and most populous city of Sierra Leone.

## 2. Results

### 2.1. Socio-Demographic Characteristics of Study Participants

A total of 394 pregnant women were enrolled in the study. Table 1 displays the characteristics of the study participants. The mean age was 24.4 ± 4.9 years and nearly half of them were in the second trimester of pregnancy (47.2%, 186/394) and primiparous (45.4%, 179/384). The majority were Muslims (73.1%, 288/394), married (78.2%, 308/394), had attained secondary level of education (223/394, 56.6%) and were employed in the informal sector (53.0%, 209/394).

### 2.2. Prevalence of HBV Status Awareness, High-Risk Practices and HBV Vaccination Status

The proportion of participants who reported that they had previously tested positive and were aware of their HBV status was low at 1.3% (5/394). Additionally, 4.1% (16/394) reported having a husband/partner with HBV (Table 1).

Of the high-risk practices explored, 76.6% (302/394) reported a history of female genital circumcision, 41.9% (165/394) had an ear piercing, 23.6% (93/394) endorsed a history of sexually transmitted infections, 22.8% (90/394) reported having had multiple sexual partners in the past, and 11.4% (45/394) had a tattoo. Furthermore, 5.1% (20/394) had a history of undergoing surgery, while 2.3% (9/394) had received a blood transfusion in the past (Table 1). Of note, only 1% (4/394) of study participants reported having received at least one dose of the HBV vaccination (Table 1).

### 2.3. Prevalence of HBsAg, HIV and HIV/HBV Co-Infection

Thirty-one of the 394 study participants tested positive for HBsAg, yielding an overall seroprevalence rate of 7.9%. The prevalence of HIV was 5.8% (23/394), while the prevalence of HIV/HBV co-infection was 0.3% (1/394) (Table 1).

The majority of participants who tested positive for HBsAg were aged 20–29 years (71.0%, 22/31), were married (74.2%, 23/31), employed (64.5%, 20/31) and in the second trimester of pregnancy (54.8%, 17/31) (Table 2).

### 2.4. Associated Factors and Predictors of HBsAg Seropositivity

Table 2 and Table 3 display the univariate and multivariate analyses of socio-demographic variables, practices and risk factors associated with HBsAg seropositivity. After controlling for confounders in the multivariate logistic regression model, having a husband/partner with HBV (aOR: 6.54; 95% CI: [1.72–24.86]; *p* = 0.006) and residing in the central part of Freetown (aOR: 4.00; 95% CI: [1.46–11.00]; *p* = 0.007) emerged as independent predictors of HBsAg seropositive status.

## 3. Discussion

To the best of our knowledge, this is the largest study to date from Sierra Leone that has assessed the prevalence and associated factors of HBsAg seropositive status among pregnant women. We observed an HBsAg prevalence rate of 7.9% among 394 pregnant women seeking antenatal care across 10 CHCs in Freetown. This figure is consistent with estimates from previous studies among non-HIV-infected pregnant women from Sierra Leone (i.e., 6% to 11%) [11,12] and approximates to the criterion for high endemicity (≥8%) [1,4]. Our findings are in agreement with multiple studies conducted in the antenatal care setting in West Africa, which have consistently reported significantly higher HBsAg seroprevalence rates than studies from other regions across SSA [17,18,19].

Additionally, our study demonstrated a high HIV prevalence of 5.8% among pregnant women. This is 3.5-fold higher than the national HIV prevalence of 1.7% and 2.6-fold higher than the HIV prevalence of 2.2% that was reported among all women in the 2019 Sierra Leone Demographic and Health Survey [13]. On the other hand, the HIV/HBV co-infection rate in this study was extremely low at 0.3%, compared with a recent study by Yendewa et al. [10], which recorded an HIV/HBV prevalence of 18.8% among HIV positive pregnant in Freetown. The low HIV/HBV prevalence among our study participants was reassuring, as current evidence suggests that HIV/HBV co-infection is associated with a high morbidity and mortality, resulting from the early onset of the acquired immune deficiency syndrome (AIDS) [20] and accelerated progression to cirrhosis, end-stage liver disease and hepatocellular carcinoma [21]. Nonetheless, our findings suggest that, similar to key populations, the parallel HIV and HBV epidemics in Sierra Leone may be disproportionately impacting pregnant women, especially in high-transmission urban settings, thus warranting a new policy focus to address this high-risk group.

Furthermore, we undertook a detailed analysis of potential risk factors traditionally attributed to HBV infection. Over three-quarters of participants reported a history of female genital circumcision, while substantially large proportions of study participants also endorsed a history of ear piercing, multiple sexual partners, sexually transmitted infections, body tattooing and surgical procedures. We found that the risk of HBsAg seropositive status did not significantly increase with any of the above-mentioned factors, which are typically associated with the horizontal transmission of HBV, nor with socio-demographic variables such as age, gender, socio-economic status, or educational attainment as previously described by studies from SSA [17,18,19]. It has been amply established that in endemic settings such as in SSA, HBV is predominantly acquired vertically through MTCT or early in life [1,3], which supports the findings of risk factor analysis in our cohort of pregnant women.

However, although most pregnant women likely acquired HBV vertically, those having a husband/partner with HBV had 6.5 times higher odds of being HBsAg seropositive compared with their counterparts who did not report having a husband/partner with HBV. This effect size, although substantially large, is likely to be an underestimate for several reasons. Firstly, information on the HBV status of the husbands/partners was collected from self-reports and not confirmed by laboratory testing. Secondly, partner testing in this setting may be low due to lack of awareness about HBV; in our cohort, only 1.3% of participants were aware of their HBV status. Thirdly, individual- and community-level stigmas related to HBV and other communicable diseases are highly prevalent and may be negatively influencing health-seeking behavior, including HBV status disclosure [22]. Nonetheless, our study is the first to document a relatively large contribution of sexual transmission in young adults of reproductive age in Sierra Leone and suggests that a mixed HBV transmission dynamic through both sexual and non-sexual contact may in fact be playing a greater role in the HBV epidemic in this hyperendemic setting than has been previously described. Interestingly, Martinson et al. [23] previously described the role of horizontal HBV transmission among children and adults in the West African country of Ghana. Similarly, Nejo et al. [24] observed a 1.7-fold higher risk of HBV among sexually active young adults in Nigeria. This highlights the need to scale up awareness-raising activities, partner screening, and treatment services to identify and link people with HBV into appropriate care.

Pregnant women living in Central Freetown accounted for only 15% of the study population; nonetheless, residence in this part of the city was associated with a 4-fold higher risk of HBV infection. While the reasons for this finding remain unclear, plausible explanations include: poor housing conditions and overcrowding in homes allowing for contact with an index HBV case [23], low socio-economic class [25], increase in commercial sexual activity [26], and lack of access to healthcare [27]. In fact, Central Freetown is densely populated and represents the hub of economic activity of the city, with limited healthcare access and poor housing conditions. Dwelling in crowded urban areas with a high prevalence of risky behaviors such as injection drug use and limited access to healthcare have been identified as major determinants of HBV infection in both developed and resource-limited regions [28,29]. Further research is needed to investigate the factors of HBV chronic carriage in this specific area of Freetown.

The HBV vaccination rate of our cohort was extremely low at 1%. Vaccination is the most effective public health intervention designed to prevent HBV infection and its clinical sequelae [1]. Despite the introduction of HBV vaccination in Sierra Leone in 2009, vaccination is not strictly enforced, and coverage has remained low among adults [13]. In three recent studies among the healthcare workers in Sierra Leone, the HBV vaccination rate ranged from 1.9% to 4.3% [30,31,32]. The universal screening of pregnant women, immunoglobulin prophylaxis and HBV vaccination for exposed newborns and adults lacking immunity could help reduce MTCT transmission rates in Sierra Leone.

Our study had a few limitations worthy of discussion. Firstly, the study was restricted to urban settings in Freetown and our observations may not be generalizable to the rest of the country. Secondly, we assessed the prevalence of HBsAg and did not undertake a complete characterization of the other sero-markers of HBV or viral-load quantification to determine active disease or prior exposure. Additionally, we were unable to verify HBV immunity through vaccination records or serological testing. Similarly, data on HIV status of participants and HBV status of their husbands/partners were collected through self-report and medical chart review, which likely resulted in an underestimate and were not confirmed with laboratory testing. Despite these limitations, our study provides a comprehensive analysis of the prevalence and associated risk factors of HBV among pregnant women that could help inform the formulation of a comprehensive national policy towards eliminating viral hepatitis as a public health threat by 2030.

## 4. Materials and Method

### 4.1. KnowHep Foundation Sierra Leone

KnowHep Foundation Sierra Leone is a non-governmental organization that has been engaged in raising awareness about viral hepatitis in Sierra Leone since 2019 through community outreach, advocacy and collaboration with partners including the Ministry of Health and Sanitation. Founded with the stated mission of improving hepatitis care delivery by reaching at least 80% of the population, KnowHep Foundation Sierra Leone offers free or low-cost services including HBV screening, treatment and monitoring of uncomplicated HBV, referrals of sicker patients for more specialized clinic- or facility-based care, and targeting high-risk groups (e.g., pregnant women and healthcare workers) for risk-reduction education and mass immunization programs.

This study was conducted using the resources of KnowHep Foundation.

### 4.2. Study Design, Population and Settings

We conducted a cross-sectional study of pregnant women aged ≥16 years who received antenatal care across 10 CHCs in Freetown, Sierra Leone, from July to September 2021. Participants were selected using convenience sampling.

Freetown is the capital and largest city of Sierra Leone. At the time of this study, the population of Freetown was estimated at 1.2 million [7]. The city is divided into the eastern, central and western districts. The eastern and central districts of Freetown are the economic and administrative hubs of the city, while the western district is largely residential.

The health needs of the residents of Freetown are served through a healthcare system that is comprised of the national referral facilities at Connaught Hospital, Ola During Children’s Hospital, Princess Christian Maternity Hospital, and several satellite clinics and peripheral health units or CHCs located throughout Freetown and its environs. The CHCs are staffed by trained community health care workers and nurses.

The study was conducted at the following 10 CHCs in Freetown, as shown in Figure 1.

Western Freetown
  1. Ogoo Farm CHC
  2. Aberdeen Maternal CHC
  3. Murray Town CHC
  4. Thompson Bay CHC
  5. Wilberforce CHC
Central Freetown
  6 Dwarzack CHC
Eastern Freetown
  7 Mabela CHC
  8 Wellington CHC
  9 Susan’s Bay CHC
  10 Old Wharf CHC

### 4.3. Participant Recruitment and Data Collection

Clinic staff and trained research personnel approached clinic attendees and explained the purpose of the study to them. Written informed consent was obtained from participants before enrollment into the study. A structured questionnaire was used to collect socio-demographic and clinical data and information on potential risk factors of HBV infection. The design of the questionnaire was informed by previous studies on this topic in Sierra Leone [10,23]. The questionnaire was piloted among 10 pregnant women to ensure the clarity of questions.

The presence of HBsAg was determined using the rapid-testing kit manufactured by Nantong Egens Diagnosis Biotechnology Co., Ltd. (Rugao, China) according to the manufacturer’s instructions. The manufacturer reported a sensitivity of 95.5% and a specificity of 98.6% [33]. HIV serostatus was based on self-reports and clinic records.

### 4.4. Statistical Analyses

Statistical analyses were performed using the SPSS Version 27.0 (Armonk, NY, USA; IBM Corp.). Categorical variables were reported as frequencies (percentages) and compared using Pearson’s chi-square or Fisher’s exact tests. Continuous variables were presented as means (standard deviation) and compared using the non-parametric independent samples Mann–Whitney U-test or Kruskal–Wallis test, as appropriate. Logistic regression was used to identify predictors of HBsAg seropositive status. Factors known to be associated with HBsAg positivity were tested in the univariate analysis. Only variables that attained a *p*-value of <0.2 in the univariate analysis were included in the multivariate regression model. Associations were reported as crude (OR) or adjusted odds ratios (aOR) with 95% confidence intervals (CI). Differences were considered statistically significant when *p* was <0.05.

### 4.5. Ethical Considerations

Clearance was obtained from the Sierra Leone Ethics Scientific and Research Committee of the Ministry of Health and Sanitation of Sierra Leone (approval date 21 July 2021). Written informed consent was obtained from all participants before enrolment into the study.

## 5. Conclusions

We observed an unacceptably high prevalence of HbsAg (7.9%) among pregnant women over a decade after HBV vaccination was introduced in Sierra Leone in 2009. Although most people acquire HBV perinatally or early in life in this hyperendemic setting, our study demonstrated that sexual transmission may be playing a more critical role in HBV acquisition in adults of reproductive age than has been previously described. Our findings support current calls advocating for scaling up HBV services to pregnant women and their sexual partners to help bring the HBV epidemic under control in Sierra Leone.

## Figures and Tables

**Table 1:** Socio-demographic and health-related characteristics of study participants (N = 394).

Characteristics	Variables	N (%)
Age, years	Mean (standard deviation)	24.4 ± 4.9
	<20	58 (14.7)
	20–29	274 (69.5)
	30–39	60 (15.2)
	40–49	2 (0.5)
Religion	Muslim	288 (73.1)
	Christian	106 (26.9)
Ethnicity	Temne	155 (39.3)
	Mende	71 (18.0)
	Limba	57 (14.5)
	Others	111 (28.2)
Relationship status	Single	82 (20.8)
	Married	308 (78.2)
	Widowed	4 (1.0)
Highest education attained	No formal education	110 (27.9)
	Primary schooling	42 (10.7)
	Secondary schooling	223 (56.6)
	Tertiary level	19 (4.8)
Area of residence in Freetown	Eastern	198 (50.3)
	Western	136 (34.5)
	Central	60 (15.2)
Employment status	Formal sector	34 (8.6)
	Informal sector	209 (53.0)
	Student	35 (8.9)
	Housewife	65 (16.5)
	Unemployed	51 (12.9)
Pregnancy stage	First trimester	45 (11.4)
	Second trimester	186 (47.2)
	Third trimester	163 (41.4)
Gravidity	First pregnancy	147 (37.3)
	Second pregnancy	152 (38.6)
	Third pregnancy	65 (16.5)
	Fourth pregnancy	22 (5.6)
	Fifth pregnancy	8 (2.0)
Parity	Nulliparous	145 (36.8)
	Primiparous	179 (45.4)
	Multiparous	70 (17.8)
Awareness of own HBV status	Yes	5 (1.3)
	No	389 (98.7)
Husband/partner with HBV	Yes	16 (4.1)
	No	378 (95.9)
History of multiple sexual partners	Yes	90 (22.8)
	No	304 (77.2)
History of any sexually transmitted infections	Yes	93 (23.6)
	No	300 (76.1)
	Unknown	1 (0.3)
Sharing toothbrush	Yes	24 (6.1)
	No	370 (93.9)
Body tattoo	Yes	45 (11.4)
	No	349 (88.6)
Ear piercing	Yes	165 (41.9)
	No	229 (58.1)
History of prior surgery	Yes	20 (5.1)
	No	374 (94.9)
History of female genital circumcision	Yes	302 (76.6)
	No	92 (23.4)
History of HBV vaccination	Yes	4 (1.0)
	No	390 (99.0)
History of abortion	Yes	39 (9.9)
	No	355 (90.1)
History of C-section	Yes	15 (3.8)
	No	379 (96.2)
History of receiving blood transfusion	Yes	9 (2.3)
	No	385 (97.7)
History of tonsillectomy	Yes	6 (1.5)
	No	388 (98.5)
Family history of liver disease	Yes	3 (0.8)
	No	391 (99.2)
HBsAg positive	Yes	31 (7.9%)
	No	363 (92.1)
HIV positive	Yes	23 (5.8)
	No	371 (94.2)
HIV/HBV co-infection	Yes	1 (0.3%)
	No	393 (99.7)

**Table 2:** Univariate analysis of factors associated with HBsAg seropositivity among pregnant women.

Characteristics	Variables	HBsAg Positive N (%)	HBsAg Negative N (%)	*p*-Value
Age, years	<20	6 (19.4)	52 (14.3)	0.628
	20–29	22 (71.0)	252 (69.4)	
	30–39	3 (9.7)	57 (15.7)	
	40–49	-	2 (0.6)	
Religion	Muslim	23 (74.2)	265 (73.0)	1.000
	Christian	8 (25.8)	98 (27.0)	
Ethnicity	Temne	12 (38.7)	143 (39.4)	0.972
	Mende	5 (16.1)	66 (18.2)	
	Limba	4 (12.9)	53 (14.6)	
	Others	10 (32.3)	101 (27.8)	
Relationship status	Single	7 (22.6)	75 (20.7)	0.353
	Married	23 (74.2)	285 (78.5)	
	Widowed	1 (3.2)	3 (0.8)	
Highest education attained	No formal education	10 (32.3)	100 (27.5)	0.399
	Primary schooling	5 (16.1)	37 (10.2)	
	Secondary schooling	14 (45.2)	209 (57.6)	
	Tertiary level	2 (6.5)	17 (4.7)	
Area of residence in Freetown	Eastern	10 (32.3)	188 (51.8)	0.040
	Western	12 (38.7)	124 (34.2)	
	Central	9 (29.0)	51 (14.0)	
Employment status	Employed	20 (64.5)	223 (61.4)	0.848
	Unemployed	11 (35.5)	140 (38.6)	
Pregnancy stage	First trimester	5 (16.1)	40 (11.0)	0.264
	Second trimester	17 (54.8)	169 (46.6)	
	Third trimester	9 (29.0)	154 (42.4)	
Gravidity	Primigravid	8 (25.8)	139 (38.3)	0.182
	Multigravid	23 (74.2)	224 (61.7)	
Parity	Nulliparous	7 (22.6)	138 (38.0)	0.152
	Primiparous	19 (61.3)	160 (44.1)	
	Multiparous	5 (16.1)	65 (17.9)	
Husband/partner with HBV	Yes	4 (12.9)	12 (3.3)	0.029
	No	27 (87.1)	351 (96.7)	
Awareness of own HBV status	Yes	-	5 (1.4)	1.000
	No	31 (100)	358 (98.6)	
HIV positive	Yes	1 (3.2)	22 (6.1)	0.518
	No	30 (96.8)	341 (93.9)	
History of multiple sexual partners	Yes	9 (29.0)	81 (22.3)	0.504
	No	22 (71.0)	282 (77.7)	
History of sexually transmitted infections	Yes	7 (22.6)	86 (23.7)	1.000
	No	24 (77.4)	277 (76.3)	
Body tattoo	Yes	3 (9.7)	42 (11.6)	0.789
	No	28 (90.3)	321 (88.4)	
Ear piercing	Yes	12 (38.7)	153 (42.1)	0.850
	No	19 (61.3)	210 (57.9)	
History of prior surgery	Yes	1 (3.2)	19 (5.2)	0.723
	No	30 (96.8)	344 (94.8)	
History of female genital circumcision	Yes	22 (71.0)	280 (77.1)	0.5061
	No	9 (29.0)	83 (22.9)	
History of abortion	Yes	3 (9.7)	36 (9.9)	1.000
	No	28 (90.3)	327 (90.1)	
History of C-section	Yes	-	15 (4.1)	0.391
	No	31 (100)	348 (95.9)	
History of receiving blood transfusion	Yes	-	9 (2.5)	0.627
	No	31 (100)	354 (97.5)	
History of tonsillectomy	Yes	-	6 (1.7)	1.000
	No	31 (100)	357 (98.3)	
Family history of liver disease	Yes	1 (3.2)	2 (0.6)	0.218
	No	30 (96.8)	361 (99.4)	

**Table 3:** Multivariate analysis of factors associated with HBsAg seropositivity among pregnant women.

Characteristics	Variables	Adjusted Odds Ratio	95% Confidence Interval	*p*-Value
Area of residence in Freetown	Eastern	Reference		0.064 0.007
	Western	2.46	0.948–6.39	
	Central	4.00	1.46–11.00	
Gravidity	Primigravid	Reference		0.786
	Multigravid	0.80	0.16–4.03	
Parity	Nulliparous	Reference		0.234 0.460
	Primiparous	2.75	0.52–14.59	
	Multiparous	2.08	0.30–14.41	
Husband/partner with HBV	Yes	6.54	1.72–24.86	0.006
	No	Reference		

## Data Availability

The data presented in this study are available on request from the corresponding author.
